# Noninferiority Study of Purified Vero Rabies Vaccine—Serum Free in 3-dose and 2-dose Preexposure Prophylaxis Regimens in Comparison With Licensed Rabies Vaccines

**DOI:** 10.1093/cid/ciae581

**Published:** 2024-11-26

**Authors:** Kulkanya Chokephaibulkit, Catherine Huoi, Terapong Tantawichien, Piroon Mootsikapun, Pope Kosalaraksa, Sasisopin Kiertiburanakul, Winai Ratanasuwan, Manuel Vangelisti, Thelma Laot, Youjun Huang, Celine Petit, Andrea-Clemencia Pineda-Peña, Carina Frago

**Affiliations:** Faculty of Medicine Siriraj Hospital, Mahidol University, Bangkok, Thailand; Clinical Development, Sanofi Vaccines, Marcy l’Etoile, France; Faculty of Medicine, Chulalongkorn University and Queen Saovabha Memorial Institute, Bangkok, Thailand; Faculty of Medicine, Srinagarind Hospital, Khon Kaen University, Khon Kaen, Thailand; Faculty of Medicine, Srinagarind Hospital, Khon Kaen University, Khon Kaen, Thailand; Faculty of Medicine Ramathibodi Hospital, Mahidol University, Bangkok, Thailand; Faculty of Medicine Siriraj Hospital, Mahidol University, Bangkok, Thailand; Development/LCM Portfolio, R&D, Sanofi Vaccines, Marcy l’Etoile, France; Clinical Development, Sanofi Vaccines, Taguig City, Philippines; Evidence Generation and Decision Sciences, Sanofi, Chengdu, China; Global Immunology, Sanofi Vaccines, Marcy l’Etoile, France; Clinical Development, Sanofi Vaccines, Marcy l’Etoile, France; Clinical Development, Sanofi Vaccines, Singapore, Singapore

**Keywords:** rabies, vaccination, preexposure prophylaxis, immunogenicity, safety

## Abstract

**Background:**

A next-generation, serum-free, highly purified Vero cell rabies vaccine, PVRV-NG2, is in development.

**Methods:**

This multicenter, observer-blind, phase 3 study evaluated the immunogenicity and safety of PVRV-NG2, compared with 2 licensed rabies vaccines (purified Vero cell rabies vaccine [PVRV] and human diploid cell vaccine [HDCV]), as a preexposure prophylaxis (PrEP) regimen. Participants were randomized 3:1:1 to PVRV-NG2, PVRV, or HDCV, as a 3-dose (cohort 1; children and adults; day [D] 0, D7, and D28) or 2-dose (cohort 2; adults; D0 and D7) PrEP regimen. The primary objective was noninferiority of PVRV-NG2 to PVRV and HDCV as 3-dose PrEP, based on the proportion of participants with rabies virus–neutralizing antibody titer ≥0.5 IU/mL at D42. Noninferiority of immune responses for 2-dose PrEP at D28 and noninferiority of 2-dose (D28) versus 3-dose (D42) HDCV were also assessed as secondary immunogenicity objectives. Safety was assessed throughout.

**Results:**

Overall, 1708 participants were enrolled (cohort 1: 505 children, 505 adults; cohort 2: 698 adults). All participants had rabies virus–neutralizing antibody titers ≥0.5 IU/mL after 3-dose PVRV-NG2 (D42), with noninferiority to PVRV and HDCV demonstrated. All secondary immunogenicity objectives were achieved, including noninferiority of 2-dose PVRV-NG2 versus 2-dose PVRV and HDCV (D28) and 3-dose HDCV (D42), and noninferiority of 2-dose HDCV versus 3-dose HDCV. The safety profile of PVRV-NG2 was comparable to those of PVRV and HDCV.

**Conclusions:**

This study supports the use of PVRV-NG2 in 2- or 3-dose PrEP regimens, with no safety concerns identified.

**Clinical Trials Registration:**

ClinicalTrials.gov identifier: NCT04127786; EudraCT: 2019-000973-22; WHO: U1111-1217-3241.

## Graphical Abstract

**Graphical Abstract:**
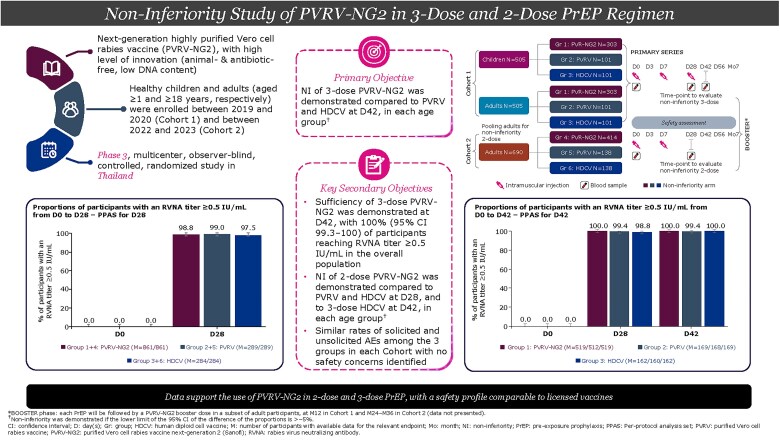

## Teaser

In this phase 3 preexposure prophylaxis study, a serum-free, next-generation purified Vero rabies vaccine, PVRV-NG2, demonstrated noninferior immunogenicity to 2 licensed vaccines as 3-dose and 2-dose primary series in pediatric and adult populations.

Rabies virus is present across most regions worldwide, although the associated burden is almost entirely in tropical countries of Asia and Africa [1, 2]. Most cases of rabies are the result of dog bites [2, 3]. Rabies is almost always fatal, and it is estimated that dog bite–related rabies causes 59 000 deaths worldwide each year [2, 3].

Potential exposure to rabies necessitates immediate postexposure prophylaxis (PEP), including vaccination [2]. A preexposure prophylaxis (PrEP) vaccination series is recommended for those at high risk of exposure to rabies because of factors such as their occupation and travel to regions where rabies is enzootic [2, 4]; in addition, PrEP should be considered for individuals living in areas where rabies is endemic and timely access to PEP limited [2]. Although PrEP does not obviate the need for PEP following suspected rabies exposure, it allows the use of a simplified PEP regimen with fewer vaccine doses and without rabies immunoglobulin [2, 4].

Purified cell culture and embryonated egg-based rabies vaccines recommended by the World Health Organization (WHO) have been available since the early 1960s [2, 5]. Currently available licensed rabies vaccines include the purified Vero cell rabies vaccine (PVRV; Verorab, Sanofi) and the human diploid cell vaccine (HDCV; Imovax Rabies, Sanofi), both of which have established safety and immunogenicity profiles and are effective in preventing symptomatic disease and death [2, 6, 7]. However, ensuring sufficient global supplies of rabies vaccines has been challenging in recent years [8, 9].

To expand vaccine supply to meet demand, a serum- and antibiotic-free, highly purified, next-generation, Vero cell rabies vaccine (PVRV-NG, Sanofi) was developed using the Pitman–Moore viral strain, which is also used for PVRV and HDCV. PVRV-NG has a lower residual DNA content (<100 pg/dose) than PVRV because of additional purification steps [10, 11]. Consistent with current WHO and European Medicines Agency recommendations, PVRV-NG is produced without raw materials derived from human or animal sources, thus minimizing the risk of contamination [10, 12]. The first formulation of PVRV-NG has already undergone extensive clinical assessment as either PrEP or PEP, with no safety concerns compared with standard-of-care rabies vaccines [10–15]. The second formulation, PVRV-NG2, includes increased antigen content compared with PVRV-NG. PVRV-NG2 was selected for phase 3 clinical trials after demonstrating similar immunogenicity and safety profiles to those of the licensed HDCV vaccine in a phase 2 dose-ranging study [13]. In this study, the highest dose PVRV-NG2 formulation was associated with higher rabies virus neutralizing antibody (RVNA) titers compared with PVRV-NG and HDCV, with a comparable proportion of participants achieving an RVNA titer ≥0.5 IU/mL to that in the HDCV group [13]; therefore, this dose was selected for phase 3 studies. PVRV-NG2 has also been evaluated in clinical trials using different PEP regimens [13, 16–18].

We undertook this pivotal study to demonstrate the noninferiority of the immune response of PVRV-NG2 versus 2 standard-of-care rabies vaccines (PVRV and HDCV) as 2- or 3-dose intramuscular PrEP regimens in children and adults, and to assess its safety profile.

## METHODS

### Study Design and Participants

This multicenter, observer-blind, controlled, randomized, phase 3 study was conducted at 4 centers in Thailand. Participants were enrolled between October 2019 and February 2020 (cohort 1) and between September 2022 and January 2023 (cohort 2). The study was conducted in accordance with ethical standards set by national and international guidelines (Supplementary Methods).

The study population included healthy children aged ≥1 year on the day of inclusion (cohort 1 only) and adults aged ≥18 years (cohorts 1 and 2) who had never received a prior rabies vaccination. Other inclusion and exclusion criteria are described in the Supplementary Materials. Eligible participants were randomized in a 3:1:1 ratio to receive PVRV-NG2, PVRV, or HDCV as a 3-dose PrEP regimen (cohort 1: groups 1, 2, and 3, respectively) or a 2-dose PrEP regimen (cohort 2: groups 4, 5, and 6, respectively). Randomization procedures are described in the Supplementary Materials.

### Interventions

PVRV-NG2, PVRV (Verorab, Sanofi), and HDCV (Imovax, Sanofi) were supplied as freeze-dried powders with diluent for reconstitution before use; both PVRV-NG2 and PVRV were reconstituted to 0.5 mL in saline and HDCV to 1 mL in water for injection. Each vaccine dose contained ≥2.5 IU inactivated rabies virus (Wistar Rabies Pitman Moore/WI 38 1503-3M strain), confirmed and released using the National Institutes of Health potency test [19]. The antigen content of each vaccine was measured by enzyme-linked immunosorbent assay (ELISA), as previously described [20, 21]. The actual antigen contents for the vaccine batches were 8.0 IU/dose for PVRV-NG2 (batch number S4497; both cohorts), 2.7 and 2.8 IU/dose for PVRV (batch numbers R1B31 [cohort 1] and U1C95 [cohort 2], respectively), and 7.3 and 8.2 IU/dose for HDCV (batch numbers P1E78 [cohort 1] and U1D92 [cohort 2], respectively).

Vaccines were administered intramuscularly in the deltoid (or anterolateral thigh for toddlers) on day (D) 0, D7, and D28 in cohort 1 and on D0 and D7 in cohort 2. Sequential vaccinations in the allocated series were done in the contralateral limb to the one previously injected.

### Immunogenicity Assessments

Blood samples for immunogenicity assessments were collected at D0 (before the first vaccination), D28 (21 days after the second vaccination), and D42 (14 days after the last vaccination) in cohort 1 and at D0 and D28 in cohort 2.

RVNA titers were measured using the rapid fluorescent focus inhibition test at the Sanofi Global Clinical Immunology Laboratory (Swiftwater, Pennsylvania, USA) [22].

### Safety Assessments

All participants were monitored for 30 minutes after each vaccination for immediate adverse events (AEs) and adverse reactions (ARs). Participants or their parents/legally acceptable representative were provided with diary cards, rulers, and thermometers and instructed to record solicited injection site reactions (pain, erythema, and swelling) and systemic reactions (fever, vomiting, crying, somnolence, decreased appetite, and irritability for participants aged ≤23 months, and fever, headache, malaise, and myalgia for those aged 2 years or older) for 7 days and unsolicited AEs/ARs for 28 days following each vaccination. AEs of special interest (anaphylaxis, encephalitis, and convulsions) and serious AEs (SAEs) occurring within at least 6 months following each vaccination were also recorded. The study investigators assigned a causal relationship to the study vaccines for each SAE.

### Statistical Analyses

A total of 1700 healthy participants were planned to be enrolled: 505 children and 505 adults in cohort 1 (303 in each age group to receive PVRV-NG2 and 101 in each age group to receive one of the control vaccines) and 690 adult participants in cohort 2 (414 to receive PVRV-NG2 and 138 each to receive one of the control vaccines). This ensured 80% power to demonstrate the primary objective and ≥80% power for each of the secondary immunogenicity objectives. Additional details on sample size and statistical power calculations are included in the Supplementary Methods and Supplementary Table 1.

The full analysis set (FAS) included all randomized participants who received at least 1 dose of the study vaccine in the primary series. Primary and secondary immunogenicity objectives for the primary vaccination series were analyzed in the per-protocol analysis set (PPAS), a subset of participants in the FAS who met all study inclusion criteria, did not meet any exclusion criteria and had no relevant protocol deviations before the time the objective was assessed (D28 or D42). Safety outcomes for each vaccine dose were assessed in the safety analysis set, which included all participants that received the dose.

The primary objective was to demonstrate that PVRV-NG2, when administered as a 3-dose PrEP regimen, was noninferior to PVRV and HDCV in each age group (children and adults), in terms of the proportion of participants achieving a RVNA titer ≥0.5 IU/mL at D42. Noninferiority was tested based on the 2-sided 95% confidence interval (CI) of the difference in proportions of participants with an RVNA titer ≥0.5 IU/mL at D42 between PVRV-NG2 and each control vaccine, separately. The 95% CIs for differences were calculated using the Wilson score method without continuity correction [23]. Noninferiority was demonstrated if the lower limit of the 95% CI was >−5% for both comparisons between PVRV-NG2 and the 2 control vaccines in each age group, respectively.

If the primary objective was met, 5 secondary immunogenicity objectives were evaluated sequentially following a fixed-sequence method [24–26], requiring each objective to be demonstrated in order to proceed to the next:

1. Sufficiency of PVRV-NG2 in the overall population (pooled children and adults) in cohort 1. Sufficiency was demonstrated if the proportion of participants with an RVNA titer ≥0.5 IU/mL at D42 was ≥99% with a lower limit of the 95% CI (using exact binomial distribution [Clopper–Pearson method]) [27] of 97% or higher.
2. Noninferiority of 2-dose PVRV-NG2 in terms of the proportion of participants (pooled cohorts 1 and 2) with RVNA titers ≥0.5 IU/mL at D28 compared with each control vaccine. Noninferiority was demonstrated using the same approach and margin as for the primary objective.
3. Noninferiority of 2-dose PVRV-NG2 at D28 (pooled cohorts 1 and 2) versus 3-dose HDCV at D42 (cohort 1) in each age group. Noninferiority of 2-dose PVRV-NG2 was demonstrated if the lower limit of the 95% CI (using the same approach as for the primary objective) of the between-group difference was >−10% (or >−5% as supplemental analysis) in both children and adults.
4. Sufficiency of 2-dose PVRV-NG2 at D28 in the overall population in pooled cohorts 1 and 2 (using the same approach as for the first secondary objective).
5. Noninferiority of 2-dose HDCV in terms of the proportion of participants with RVNA titers ≥0.5 IU/mL at D28 compared to 3-dose HDCV at D42 in pooled children and adults in cohort 1. Noninferiority of 2-dose HDCV was demonstrated if the lower limit of the 95% CI of the between-group difference was >−10% (or >−5% as supplemental analysis).

All other immunogenicity and safety analyses were descriptive. All statistical analyses were performed using SAS, version 9.4 or later (SAS Institute, Cary, North Carolina, USA).

## RESULTS

### Participants

A total of 1708 participants were enrolled and randomized to receive a 3-dose primary vaccination series (cohort 1; 505 adults and 505 children) or 2-dose primary vaccination series (cohort 2; 698 adults). The numbers of participants randomized to each group and included in each analysis set are shown in Supplementary Table 2. The flow of participants through the study following the primary vaccination series is summarized in Supplementary Figure 1.

Baseline characteristics of the participants are summarized in Table 1. All participants were of Asian origin and approximately two-thirds were female.

**Table 1.** Summary of Participant Characteristics at Baseline by Randomized Group (FAS)

		Cohort 1 (3-dose)			Cohort 2 (2-dose)		
Age Group		Group 1 PVRV-NG2 (N = 607)	Group 2 PVRV (N = 202)	Group 3 HDCV (N = 200)	Group 4 PVRV-NG2 (N = 419)	Group 5 PVRV (N = 139)	Group 6 HDCV (N = 139)
Overall	Age, y						
	M	607	202	200	419	139	139
	Mean (SD)	22.9 (17.1)	22.6 (16.1)	22.4 (16.1)	37.4 (11.4)	38.4 (11.1)	37.3 (10.9)
	Min; max	1; 78	1; 70	1; 71	18; 67	18; 65	18; 63
	Median	17.0	21.0	18.5	37.0	39.0	38.0
	Age group, n (%)						
	Children (<18 y)	305 (50.2)	99 (49.0)	100 (50.0)	0	0	0
	12–23 m	17 (2.8)	2 (1.0)	2 (1.0)	0	0	0
	2–11 y	198 (32.6)	70 (34.7)	68 (34.0)	0	0	0
	12–17 y	90 (14.8)	27 (13.4)	30 (15.0)	0	0	0
	Adult (≥18 y)	302 (49.8)	103 (51.0)	100 (50.0)	419 (100)	139 (100)	139 (100)
	18–40 y	192 (31.6)	68 (33.7)	69 (34.5)	262 (62.5)	78 (56.1)	82 (59.0)
	41–64 y	100 (16.5)	33 (16.3)	29 (14.5)	153 (36.5)	58 (41.7)	57 (41.0)
	≥65 y	10 (1.6)	2 (1.0)	2 (1.0)	4 (1.0)	3 (2.2)	0
	Sex, n (%)						
	Male	208 (34.3)	56 (27.7)	78 (39.0)	154 (36.8)	43 (30.9)	41 (29.5)
	Female	399 (65.7)	146 (72.3)	122 (61.0)	265 (63.2)	96 (69.1)	98 (70.5)
	Racial origin, n (%)						
	Asian	607 (100)	202 (100)	200 (100)	419 (100)	139 (100)	139 (100)
	Weight, kg						
	M	607	202	200	419	139	139
	Mean (SD)	47.35 (21.36)	47.63 (21.42)	48.38 (22.84)	67.75 (17.04)	66.58 (15.81)	66.98 (18.01)
	Min; max	8.0; 113.5	9.0; 119.0	10.0; 130.0	40.6; 140.2	40.4; 138.4	39.3; 153.9
	Median	50.00	50.05	49.35	64.90	64.80	61.80
	Height, cm						
	M	607	202	200	419	139	139
	Mean (SD)	143.8 (23.9)	143.8 (22.3)	144.9 (24.1)	162.8 (8.5)	162.5 (8.5)	161.8 (8.1)
	Min; max	75; 185	77; 179	82; 180	145; 187	140; 192	148; 185
	Median	153.0	153.0	154.0	161.0	160.0	160.0
	BMI, kg/m^2^						
	M	607	202	200	419	139	139
	Mean (SD)	21.38 (5.53)	21.54 (5.67)	21.38 (5.51)	25.45 (5.45)	25.09 (5.04)	25.43 (5.94)
	Min; max	11.9; 43.8	11.3; 37.1	13.2; 42.4	14.9; 48.5	16.6; 45.2	16.8; 58.6
	Median	20.80	21.25	20.70	24.50	24.20	24.10
Children (<18 y)	Age, y						
	M	305	99	100	-	-	-
	Mean (SD)	8.6 (4.5)	8.6 (4.0)	8.6 (4.3)	-	-	-
	Min; max	1; 17	1; 17	1; 17	-	-	-
	Median	8.0	8.0	8.0	-	-	-
	Sex, n (%)						
	Male	146 (47.9)	41 (41.4)	54 (54.0)	-	-	-
	Female	159 (52.1)	58 (58.6)	46 (46.0)	-	-	-
	Racial origin, n (%)						
	Asian	305 (100)	99 (100)	100 (100)	-	-	-
	Weight, kg	…					
	M	305	99	100	…	…	…
	Mean (SD)	32.69 (17.58)	31.16 (15.31)	33.57 (20.67)	…	…	…
	Min; max	8.0; 97.0	9.0; 77.5	10.0; 130.0	…	…	
	Median	28.30	25.50	27.90	…	…	…
	Height, cm	…					
	M	305	99	100	…	…	…
	Mean (SD)	128.7 (25.0)	128.7 (22.7)	129.1 (24.4)	…	…	…
	Min; max	75; 180	77; 177	82; 175	…	…	…
	Median	129.0	125.0	127.5	…	…	…
	BMI, kg/m^2^	…					
	M	305	99	100	…	…	…
	Mean (SD)	18.23 (4.32)	17.64 (3.73)	18.36 (4.87)	…	…	…
	Min; max	11.9; 35.2	11.3; 28.8	13.2; 42.4	…	…	…
	Median	16.80	16.70	17.10	…	…	…
Adults (≥18 y)	Age, y						
	M	302	103	100	419	139	139
	Mean (SD)	37.3 (12.3)	36.1 (11.0)	36.2 (10.7)	37.4 (11.4)	38.4 (11.1)	37.3 (10.9)
	Min; max	18; 78	20; 70	20; 71	18; 67	18; 65	18; 63
	Median	35.5	35.0	34.5	37.0	39.0	38.0
	Sex, n (%)						
	Male	62 (20.5)	15 (14.6)	24 (24.0)	154 (36.8)	43 (30.9)	41 (29.5)
	Female	240 (79.5)	88 (85.4)	76 (76.0)	265 (63.2)	96 (69.1)	98 (70.5)
	Racial origin, n (%)						
	Asian	302 (100)	103 (100)	100 (100)	419 (100)	139 (100)	139 (100)
	Weight, kg						
	M	302	103	100	419	139	139
	Mean (SD)	62.15 (12.98)	63.47 (12.76)	63.20 (13.34)	67.75 (17.04)	66.58 (15.81)	66.98 (18.01)
	Min; max	38.0; 113.5	42.0; 119.0	42.0; 101.7	40.6; 140.2	40.4; 138.4	39.3; 153.9
	Median	60.00	62.40	60.85	64.90	64.80	61.80
	Height, cm						
	M	302	103	100	419	139	139
	Mean (SD)	159.0 (7.5)	158.4 (7.0)	160.7 (8.0)	162.8 (8.5)	162.5 (8.5)	161.8 (8.1)
	Min; max	140; 185	145; 179	144; 180	145; 187	140; 192	148; 185
	Median	158.0	158.0	159.0	161.0	160.0	160.0
	BMI, kg/m^2^						
	M	302	103	100	419	139	139
	Mean (SD)	24.57 (4.73)	25.28 (4.59)	24.40 (4.35)	25.45 (5.45)	25.09 (5.04)	25.43 (5.94)
	Min; max	15.6; 43.8	16.0; 37.1	16.4; 35.5	14.9; 48.5	16.6; 45.2	16.8; 58.6
	Median	24.10	25.10	23.30	24.50	24.20	24.10

For the overall age group, percentages are based on N; for individual age groups, percentages are based on M.

Abbreviations: BMI, body mass index; FAS, full analysis set; HDCV, human diploid cell vaccine; M, number of participants with available data for the relevant parameter; N, number of participants in FAS; PVRV, purified Vero cell rabies vaccine; PVRV-NG2, next-generation purified Vero cell rabies vaccine; SD, standard deviation.

### Immunogenicity Outcomes

#### Noninferiority of 3-dose PVRV-NG2 (Group 1) Versus 3-dose PVRV (Group 2) and HDCV (Group 3)

The proportion of participants with RVNA titers ≥0.5 IU/mL at D42 following the 3-dose primary vaccination series (PPAS for D42) is summarized by vaccine group and age group in Table 2. The primary objective of noninferiority of PVRV-NG2 (group 1) versus PVRV (group 2) and versus HDCV (group 3) at D42 was demonstrated in children and adults, with lower limits of the 95% CIs for each comparison higher than the −5% margin (Table 2).

**Table 2.** Noninferiority of the 3-dose Primary Series With PVRV-NG2 Versus the 2 Control Vaccines at D42: Proportion of Participants Achieving RVNA Titers ≥0.5 IU/mL in Cohort 1 (PPAS for D42)

Age Group	Group 1 (PVRV-NG2) (N = 519)			Group 2 (PVRV) (N = 169)			Group 3 (HDCV) (N = 162)			Group 1—Group 2			Group 1—Group 3		
	n/M	%	95% CI	n/M	%	95% CI	n/M	%	95% CI	Difference (%)	95% CI	Noninferiority	Difference (%)	95% CI	Noninferiority
Children (<18 y)	265/265	100	(98.6–100)	85/85	100	(95.8–100)	83/83	100	(95.7–100)	0	(−1.4 to 4.3)	Yes	0	(−1.4 to 4.4)	Yes
Adults (≥18 y)	254/254	100	(98.6–100)	83/84	98.8	(93.5–100)	79/79	100	(95.4–100)	1.2	(−.6 to 6.4)	Yes	0	(−1.5 to 4.6)	Yes

The 95% CIs for single proportions were calculated from the exact binomial method; 95% CIs for the difference of the proportions between groups were calculated from the Wilson score method without continuity correction. Each noninferiority was demonstrated if the lower limit of the 95% CI of the difference between the test group (group 1) and control group (group 2 or group 3) was >−5% at D42. The overall noninferiority between PVRV-NG2 and comparator vaccines at D42 was demonstrated if noninferiority between gbroup 1 and group 2 and between group 1 and group 3 were both demonstrated at D42 in each age group.

Abbreviations: CI, confidence interval; D, day; HDCV, human diploid cell vaccine; M, number of participants with available data for the endpoint; n, number of participants with RVNA titer ≥0.5 IU/mL; N, number of participants in PPAS for D42; PPAS, per-protocol analysis set; PVRV, purified Vero cell rabies vaccine; PVRV-NG2, next-generation purified Vero cell rabies vaccine; RVNA, rabies virus neutralizing antibody.

#### Sufficiency of 3-dose PVRV-NG2 (Group 1)

The sufficiency of PVRV-NG2 (group 1) at D42 was demonstrated based on the PPAS for D42: 100% of participants who received 3 doses of PVRV-NG2 achieved an RVNA titer ≥0.5 IU/mL with the lower limit of the 95% CI (99.3–100), thereby achieving the ≥97% margin for sufficiency.

#### Noninferiority of 2-dose PVRV-NG2 (Groups 1 and 4) Versus 2-dose PVRV (Groups 2 and 5) and HDCV (Groups 3 and 6)

Noninferiority of 2-dose PVRV-NG2 (groups 1 and 4) versus 2-dose PVRV (groups 2 and 5) and HDCV (groups 3 and 6) at D28 was demonstrated based on the PPAS for D28 in both children and adults, as indicated by lower limits of the 95% CIs for each comparison that were greater than the −5% margin (Table 3).

**Table 3.** Noninferiority of 2-dose PVRV-NG2 Versus the 2 Control Vaccines at D28: Proportion of Participants Achieving RVNA Titers ≥0.5 IU/mL in Cohorts 1 and 2 Combined (PPAS for D28)

Age Group	Groups 1 + 4 (PVRV-NG2) (N = 861)			Groups 2 + 5 (PVRV) (N = 289)			Groups 3 + 6 (HDCV) (N = 284)			(Groups 1 + 4)—(Groups 2 + 5)			(Groups 1 + 4)—(Groups 3 + 6)		
	n/M	%	95% CI	n/M	%	95% CI	n/M	%	95% CI	Difference (%)	95% CI	Noninferiority	Difference (%)	95% CI	Noninferiority
Children (<18 y)	266/266	100	(98.6–100)	86/86	100	(95.8–100)	81/81	100	(95.5–100)	0	(−1.4 to 4.3)	Yes	0	(−1.4 to 4.5)	Yes
Adults (≥18 y)	585/595	98.3	(96.9–99.2)	200/203	98.5	(95.7–99.7)	196/203	96.6	(93.0–98.6)	−0.2	(−1.9 to 2.7)	Yes	1.8	(−0.5 to 5.3)	Yes

The 95% CIs for single proportions were calculated from the exact binomial method; 95% CIs for the difference between groups were calculated from the Wilson score method without continuity correction. Each noninferiority was demonstrated if the lower limit of the 95% CI of the difference of the proportions between the test group (groups 1 + 4) and control group (groups 2 + 5, groups 3 + 6) was >−5% at D28. If the sufficiency objective for PVRV-NG2 was reached at D42, the overall noninferiority between PVRV-NG2 and the comparator vaccines at D28 was demonstrated if noninferiority between pooled groups 1 + 4 and pooled groups 2 + 5, and between pooled groups 1 + 4 and pooled groups 3 + 6, were both demonstrated at D28 in each age group.

Abbreviations: CI, confidence interval; D, day; HDCV, human diploid cell vaccine; M, number of participants with available data for the endpoint; n, number of participants with RVNA titer ≥0.5 IU/mL; N, number of participants in PPAS for D28; PPAS, per-protocol analysis set; PVRV, purified Vero cell rabies vaccine; PVRV-NG2, next-generation purified Vero cell rabies vaccine; RVNA, rabies virus neutralizing antibody.

#### Noninferiority of 2-dose PVRV-NG2 (Groups 1 and 4) Versus 3-dose HDCV (Group 3)

The noninferiority of 2-dose PVRV-NG2 (groups 1 and 4) at D28 (in the PPAS for D28) versus 3-dose HDCV (group 3) at D42 (in the PPAS for D42) was demonstrated for both children and adults, with lower limits of 95% CIs greater than both the −10% and −5% noninferiority margins (Table 4).

**Table 4.** Noninferiority of 2-dose PVRV-NG2 (D28) Versus 3-dose HDCV (D42): Proportion of Participants Achieving RVNA Titers ≥0.5 IU/mL in Cohorts 1 and 2 Combined (PPAS)

Age Group	Groups 1 + 4 (PVRV-NG2) at D28 (N = 861)			Group 3 (HDCV) at D42 (N = 162)			(Groups 1 + 4) at D28—Group 3 at D42			
	n/M	%	95% CI	n/M	%	95% CI	Difference (%)	95% CI	Noninferiority (Margin, −10%)	Noninferiority (Margin, −5%)**^a^**
Children (<18 y)	266/266	100	(98.6–100)	83/83	100	(95.7–100)	0	(−1.4 to 4.4)	Yes	Yes
Adults (≥18 y)	585/595	98.3	(96.9–99.2)	79/79	100	(95.4–100)	−1.7	(−3.1 to 3.0)	Yes	Yes

The 95% CIs for single proportions were calculated using the exact binomial method; 95% CIs for the difference between groups were calculated using the Wilson score method without continuity correction. Noninferiority was demonstrated if the lower limit of the 95% CI of the difference of the proportions between 2-dose PVRV-NG2 at D28 and 3-dose HDCV at D42 was >−10%. If the noninferiority objective for PVRV-NG2 versus comparator vaccines at D28 was demonstrated, the overall noninferiority of 2-dose PVRV-NG2 at D28 versus 3-dose HDCV at D42 was demonstrated if the noninferiority between pooled groups 1 + 4 and group 3 was both demonstrated in each age group.

Abbreviations: CI, confidence interval; D, day; HDCV, human diploid cell vaccine; M, number of participants with available data for the endpoint; n, number of participants with RVNA titer ≥0.5 IU/mL; N, number of participants in PPAS for D28 for 2-dose PVRV-NG2 (groups 1 + 4) and number of participants in PPAS for D42 for 3-dose HDCV (group 3); PPAS, per-protocol analysis set; PVRV-NG2, next-generation purified Vero cell rabies vaccine; RVNA, rabies virus neutralizing antibody.

^a^Supplemental analysis was based on noninferiority margin of −5% using similar methodology.

#### Sufficiency of 2-dose PVRV-NG2 (Groups 1 and 4)

In the PPAS for D28, the proportion of participants who received 2 doses of PVRV-NG2 (groups 1 and 4) and had an RVNA titer ≥0.5 IU/mL at D28 was 98.8% (95% CI, 97.9–99.4). Although the point estimation was numerically slightly lower than the predefined estimation of 99%, the lower limit of the 95% CI exceeded the sufficiency margin of 97% for the lower bound. Thus, the sufficiency of PVRV-NG2 was considered to have been met from a clinical standpoint.

#### Noninferiority of 2-dose HDCV (Group 3) Versus 3-dose HDCV (Group 3)

The noninferiority of 2-dose HDCV at D28 (in the PPAS for D28) versus 3-dose HDCV at D42 (in the PPAS for D42) was demonstrated, with a lower limit of the 95% CI for differences greater than both the −10% and −5% noninferiority margins (Table 5).

**Table 5.** Noninferiority of 2-dose HDCV (D28) Versus 3-dose HDCV (D42): Proportion of Participants Achieving RVNA Titers ≥0.5 IU/mL in Cohort 1 (PPAS)

Group 3 (HDCV) at D28 (N = 160)			Group 3 (HDCV) at D42 (N = 162)			Group 3 at D28—Group 3 at D42			
n/M	%	95% CI	n/M	%	95% CI	Difference (%)	95% CI: Mover Wilson score	Noninferiority (Margin, −10%)	Noninferiority (Margin, −5%)^a^
158/160	98.8	(95.6–99.8)	160/160	100	(97.7–100)	−1.3	(−4.4 to 1.3)	Yes	Yes

The 95% CIs for single proportions were calculated using the exact binomial method; 95% CIs for differences were calculated using the GLM for repeated measured data with categorical response under binomial distribution (link function = identity) or the Mover Wilson score method if GLM did not converge. Each noninferiority was demonstrated if the lower limit of the 95% CI of the difference of the proportions between the 2-dose HDCV at D28 and 3-dose HDCV at D42 was >−10%. If the sufficiency objective of PVRV-NG2 was reached at D28, the noninferiority of 2-dose HDCV at D28 versus 3-dose HDCV at D42 was demonstrated if the lower limit of the 95% CI of the difference of the proportions between the 2-dose HDCV at D28 and 3-dose HDCV at D42 was >−10%.

Abbreviations: CI, confidence interval; D, day; GLM, generalized linear model; HDCV, human diploid cell vaccine; M, number of participants with available data for endpoint at both D28 and D42; n, number of participants with RVNA titer ≥ 0.5 IU/mL and available data at both D28 and D42; N, number of participants in PPAS; PPAS, per-protocol analysis set; PVRV-NG2, next-generation purified Vero cell rabies vaccine; RVNA, rabies virus neutralizing antibody.

^a^Supplemental analysis was based on a noninferiority margin of −5% using similar methodology.

#### RVNA Titers at D0, D28, and D42

**Figure 1.**
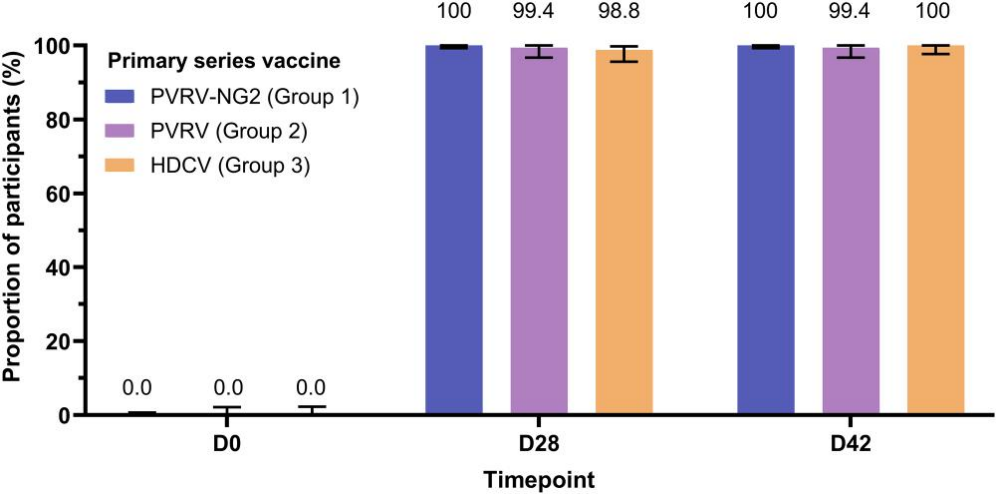
Proportion of participants (95% CI) with RVNA titer ≥0.5 IU/mL with the 3-dose PrEP primary series (cohort 1, overall population, pooled children and adults) (PPAS). CI, confidence interval; D, day; HDCV, human diploid cell vaccine; PPAS, per-protocol analysis set; PrEP, preexposure prophylaxis; PVRV, purified Vero cell rabies vaccine; PVRV-NG2, next-generation purified Vero cell rabies vaccine; RVNA, rabies virus neutralizing antibody.

**Figure 2.**
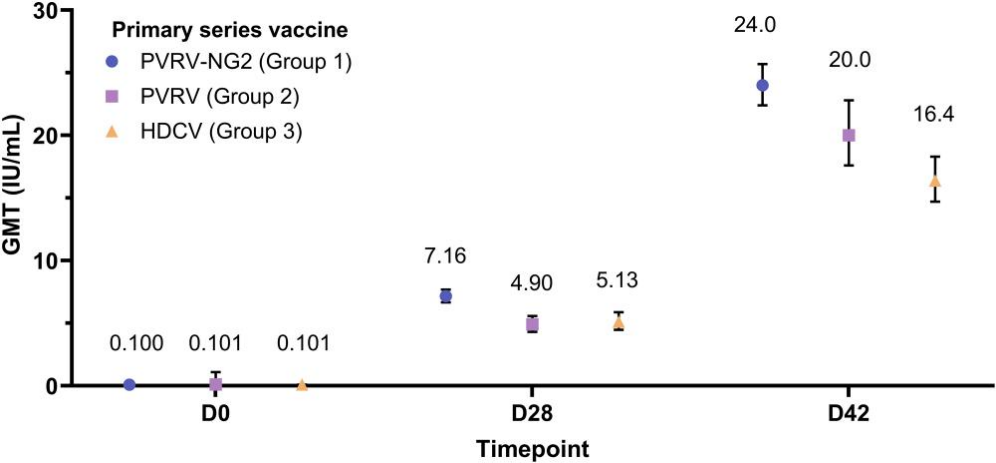
RVNA GMT (95% CI) in participants receiving the 3-dose PrEP primary series (cohort 1, overall population, pooled children and adults) (PPAS). CI, confidence interval; D, day; GMT, geometric mean titer; HDCV, human diploid cell vaccine; PPAS, per-protocol analysis set; PrEP, preexposure prophylaxis; PVRV, purified Vero cell rabies vaccine; PVRV-NG2, next-generation purified Vero cell rabies vaccine; RVNA, rabies virus neutralizing antibody.

No participant in any group had an RVNA titer ≥0.5 IU/mL at D0 (baseline). In cohort 1, by D28 and D42 after vaccination, the proportions increased to ≥98.8% and ≥99.4%, respectively (Figure 1). In cohort 2, >96.0% of participants had RVNA titer ≥0.5 IU/mL at D28 (Supplementary Figure 2). RVNA geometric mean titers are summarized in Figure 2 and Supplementary Table 3 (cohort 1) and Supplementary Figure 3 and Supplementary Table 4 (cohort 2).

### Safety

No immediate unsolicited AEs or ARs were reported within 30 minutes of any injection in any group in either cohort. The overall safety profile of PVRV-NG2 was comparable to those of PVRV and HDCV (Table 6 and Supplementary Table 5). Most solicited ARs within 7 days after any injection were of grade 1 or 2 intensity and resolved spontaneously within 1–3 days.

**Table 6.** Safety Overview After any Primary Series Vaccinations in Cohort 1 (3-dose, Children and Adults) and Cohort 2 (2-dose, Adults) in All Age Groups (SafAS)

Participants in Cohort 1 Experiencing at Least One Event	Group 1 3-dose PVRV-NG2 (N = 607)			Group 2 3-dose PVRV (N = 202)			Group 3 3-dose HDCV (N = 200)		
	n/M	%	(95% CI)	n/M	%	(95% CI)	n/M	%	(95% CI)
Within 30 min after any vaccine injections in the primary series									
Immediate unsolicited AE	0/607	0	(0; 0.6)	0/202	0	(0; 1.8)	0/200	0	(0; 1.8)
Immediate unsolicited AR	0/607	0	(0; 0.6)	0/202	0	(0; 1.8)	0/200	0	(0; 1.8)
Solicited reaction within 7 d after any vaccine injections in the primary series	406/607	66.9	(63.0; 70.6)	118/201	58.7	(51.6; 65.6)	126/200	63.0	(55.9; 69.7)
Grade 3	21/607	3.5	(2.2; 5.2)	3/201	1.5	(0.3; 4.3)	1/200	0.5	(0; 2.8)
Solicited injection site reaction	348/607	57.3	(53.3; 61.3)	98/201	48.8	(41.7; 55.9)	107/200	53.5	(46.3; 60.6)
Grade 3	5/607	0.8	(0.3; 1.9)	1/201	0.5	(0; 2.7)	0/200	0	(0; 1.8)
Solicited systemic reaction	313/607	51.6	(47.5; 55.6)	88/201	43.8	(36.8; 50.9)	93/200	46.5	(39.4; 53.7)
Grade 3	19/607	3.1	(1.9; 4.8)	2/201	1.0	(0.1; 3.5)	1/200	0.5	(0; 2.8)
Up to 28 d after any vaccine injections in the primary series									
Unsolicited AE	137/607	22.6	(19.3; 26.1)	45/202	22.3	(16.7; 28.6)	40/200	20.0	(14.7; 26.2)
Unsolicited AR	1/607	0.2	(0; 0.9)	0/202	0	(0; 1.8)	0/200	0	(0; 1.8)
Unsolicited injection site AR	1/607	0.2	(0; 0.9)	0/202	0	(0; 1.8)	0/200	0	(0; 1.8)
Unsolicited systemic AE	136/607	22.4	(19.1; 25.9)	45/202	22.3	(16.7; 28.6)	40/200	20.0	(14.7; 26.2)
Unsolicited systemic AR	0/607	0	(0; 0.6)	0/202	0	(0; 1.8)	0/200	0	(0; 1.8)
During the primary series active period^a^									
SAE	5/607	0.8	(0.3; 1.9)	1/202	0.5	(0; 2.7)	1/200	0.5	(0; 2.8)
AESI	0/607	0	(0; 0.6)	0/202	0	(0; 1.8)	0/200	0	(0; 1.8)
During the whole primary series^b^									
SAE	10/607	1.6	(0.8; 3.0)	3/202	1.5	(0.3; 4.3)	5/200	2.5	(0.8; 5.7)
AESI	0/607	0	(0; 0.6)	0/202	0	(0; 1.8)	0/200	0	(0; 1.8)

Participants in Cohort 2 Experiencing at Least One Event	Group 4 2-dose PVRV-NG2 (N = 419)			Group 5 2-dose PVRV (N = 139)			Group 6 2-dose HDCV (N = 139)		
	n/M	%	(95% CI)	n/M	%	(95% CI)	n/M	%	(95% CI)
Within 30 min after any vaccine injections in the primary series									
Immediate unsolicited AE	0/419	0	(0; 0.9)	0/139	0	(0; 2.6)	0/139	0	(0; 2.6)
Immediate unsolicited AR	0/419	0	(0; 0.9)	0/139	0	(0; 2.6)	0/139	0	(0; 2.6)
Solicited reaction within 7 d after any vaccine injections in the primary series	128/419	30.5	(26.2; 35.2)	33/139	23.7	(16.9; 31.7)	47/139	33.8	(26.0; 42.3)
Grade 3	2/419	0.5	(0.1; 1.7)	0/139	0	(0, 2.6)	0/139	0	(0; 2.6)
Solicited injection site reaction	111/419	26.5	(22.3; 31.0)	30/139	21.6	(15.1; 29.4)	43/139	30.9	(23.4; 39.3)
Grade 3	0/419	0	(0; 0.9)	0/139	0	(0; 2.6)	0/139	0	(0; 2.6)
Solicited systemic reaction	94/419	22.4	(18.5; 26.7)	16/139	11.5	(6.7; 18.0)	33/139	23.7	(16.9; 31.7)
Grade 3	2/419	0.5	(0.1; 1.7)	0/139	0	(0; 2.6)	0/139	0	(0; 2.6)
Up to 28 d after any vaccine injections in the primary series									
Unsolicited AE	44/419	10.5	(7.7; 13.8)	18/139	12.9	(7.9; 19.7)	12/139	8.6	(4.5; 14.6)
Unsolicited AR	1/419	0.2	(0; 1.3)	0/139	0	(0; 2.6)	1/139	0.7	(0; 3.9)
Unsolicited injection site AR	1/419	0.2	(0; 1.3)	0/139	0	(0; 2.6)	1/139	0.7	(0; 3.9)
Unsolicited systemic AE	43/419	10.3	(7.5; 13.6)	18/139	12.9	(7.9; 19.7)	11/139	7.9	(4.0; 13.7)
Unsolicited systemic AR	0/419	0	(0; 0.9)	0/139	0	(0; 2.6)	0/139	0	(0; 2.6)
During the primary series active period^a^									
SAE	0/419	0	(0; 0.9)	0/139	0	(0; 2.6)	0/139	0	(0; 2.6)
AESI	0/419	0	(0; 0.9)	0/139	0	(0; 2.6)	0/139	0	(0; 2.6)
During the whole primary series^b^									\
SAE	0/419	0	(0; 0.9)	0/139	0	(0; 2.6)	0/139	0	(0; 2.6)
AESI	0/419	0	(0; 0.9)	0/139	0	(0; 2.6)	0/139	0	(0; 2.6)

ARs were defined as those reported by a study investigator as related to study vaccine. If the relationship to study vaccine (investigational product) was missing, the event was also considered to be related.

Percentages were based on M of each age group.

Abbreviations: AE, adverse event; AESI, AE of special interest; AR, adverse reactions related to study vaccine; CI, confidence interval; D, day; HDCV, human diploid cell vaccine; M, number of participants with available data for the relevant endpoint; n, number of participants experiencing the endpoint listed; N, number of participants in the SafAS; PVRV, purified Vero cell rabies vaccine; PVRV-NG2, next-generation purified Vero cell rabies vaccine; SAE, serious AE; SafAS, safety analysis set.

^a^From D0 to 28 d after the last vaccination in the primary series.

^b^From D0 through the end of the 6-m follow-up period after the last vaccination in the primary series.

Three participants experienced AEs that led to discontinuation from the study during the primary vaccination series, all of which were due to suspected exposure to rabies from animal bites and not related to the study intervention. These comprised 1 (0.2%) child in the PVRV-NG2 group in cohort 1 (dog bite, recovered) and 2 adults in cohort 2, 1 (0.2%) in the PVRV-NG2 group and 1 (0.7%) in the HDCV group (both cat bites, with outcomes unknown/not reported).

In cohort 1, between D0 and the end of the 6-month safety follow-up postvaccination, the proportion of participants who experienced at least 1 SAE was 1.6% (10/607) in the PVRV-NG2 group, 1.5% (3/202) in the PVRV group, and 2.5% (5/200) in the HDCV group. None of the SAEs were related to the study vaccine. No participants in cohort 2 reported SAEs. No AEs of special interest or deaths were reported in either cohort.

## DISCUSSION

This pivotal phase 3 study demonstrated that the immune response after the 3-dose PVRV-NG2 PrEP regimen was noninferior to that achieved after the 3-dose regimens of 2 control vaccines, PVRV and HDCV, in both children and adults in terms of the proportion of participants who achieved RVNA titer ≥0.5 IU/mL. No safety issues were identified with any of the vaccines. All key secondary immunogenicity endpoints were met.

The immune response with the 3-dose PVRV-NG2 PrEP regimen, as indicated by the proportion of participants with RVNA titer ≥0.5 IU/mL and RVNA geometric mean titers at D42, was also comparable to that seen with PVRV and HDCV vaccines in previous studies that compared these licensed vaccines with PVRV-NG [10, 11, 15]. Furthermore, the immune response with PVRV-NG2 in the current study appears to have been improved compared to that reported for the initial PVRV-NG formulation in these studies [11, 15]. The comparable immunogenicity profile of PVRV-NG2 as part of PEP in combination with HRIG has also been confirmed, relative to HDCV [13] and relative to PVRV and HDCV [18].

In this study, we assessed both the 3-dose and 2-dose PrEP regimens, to be consistent with clinical practice at the time the study was initiated and recommendations by the WHO (2-dose) [2] and the US Advisory Committee on Immunization Practices (3-dose prior to updated recommendations for 2-dose PrEP in 2022) [4]. A 2-dose PrEP regimen is also recommended by other countries [28–32]. We confirmed directly that 2-dose PrEP (PVRV-NG2 or HDCV) was noninferior to 3-dose PrEP (HDCV).

This study will also evaluate the immunogenicity and safety of PVRV-NG2 as a booster vaccination in subsets of participants from each cohort, regardless of which vaccine they received for the primary series. The PVRV-NG2 booster vaccinations will be administered 1 year after the first vaccination in cohort 1 and between 2 and 3 years after the first vaccination in cohort 2. Results for the immunogenicity and safety of PVRV-NG2 as a booster vaccination following the PrEP primary series in these 2 subsets will be reported in a later publication.

Limitations of our study include the different recruitment time periods for cohort 1 and cohort 2 and that the study was conducted in only 1 country, with cohort 2 recruited at a single center. However, there were no notable differences in immune response (proportion of participants reaching RVNA titers ≥0.5 IU/mL) per group across different study centers in cohort 1 (data not shown). In addition, very few participants younger than age 2 years and older than age 65 years were recruited.

The safety profile of PVRV-NG2 in this study was broadly similar to that previously reported for PVRV-NG when given as PrEP in children and adults [10, 11, 15]. Furthermore, we also showed that the safety profile of PVRV-NG2 was comparable to that of PVRV and HDCV.

In conclusion, this study supports the use of PVRV-NG2 in 3-dose or 2-dose PrEP regimens, with no safety concerns identified in any age group.

## Supplementary Material

ciae581_Supplementary_Data

## Supplementary Data

Supplementary materials are available at *Clinical Infectious Diseases* online. Consisting of data provided by the authors to benefit the reader, the posted materials are not copyedited and are the sole responsibility of the authors, so questions or comments should be addressed to the corresponding author.

## Notes

***Author contributions*.** T. L., A.-C. P.-P., and C. P. contributed to the concept or design of the study; K. C., T. T., P. M., P. K., S. K., W. R., T. L., A.-C. P.-P., and C. P. were involved in data acquisition; and all authors were involved in the analysis or interpretation of the data. C. H., Y. H., C. P., and C. F. were involved in drafting the initial manuscript. All authors critically revised the manuscript, approved the final version for publication, and are accountable for its accuracy and integrity.

***Acknowledgments*.** The authors thank all participants who volunteered to take part in the study; the investigators and staff at study sites; and the staff who coordinated this study. The authors thank Yuvadee Yaiprayoon, Mae Ann Verdan, Qian Jiang, Emilia Jordanov, Ada-Maria Minutello, Elisa Valero, Florence Coux, Auriane Nadal, Julie Supah, Min Yao, and Adeline Pallez of Sanofi for their contributions to the study design and conduct, and to data interpretation. Editorial assistance with the preparation of the manuscript was provided by Raewyn M. Poole and Richard Glover of inScience Communications, Springer Healthcare, funded by Sanofi. The authors also thank Isabel Gregoire for editorial assistance and manuscript coordination on behalf of Sanofi.

***Financial support***. This work was supported by Sanofi, United States.

***Potential conflicts of interest***. C. H., M. V., Y. H., C. P., A.-C. P.-P., and C. F. are Sanofi employees and may hold shares. T. L. was employed by Sanofi at the time of the study. K. C. has received support for this study from Sanofi. P. K. has received research funding from Sanofi through Khon Kaen University. All other authors report no potential conflicts.

***Availability of data and materials*.** Qualified researchers may request access to patient-level data and related documents (including, eg, the clinical study report, study protocol with any amendments, blank case report form, statistical analysis plan, dataset specifications). Patient-level data will be anonymized, and study documents will be redacted to protect the privacy of trial participants. Further details on Sanofi's data sharing criteria, eligible studies, and process for requesting access can be found at https://vivli.org/.
