# Supplementary material for: Noninferiority Study of Purified Vero Rabies Vaccine—Serum Free in 3-dose and 2-dose Preexposure Prophylaxis Regimens in Comparison With Licensed Rabies Vaccines
Source: Clin Infect Dis. 2024 Nov 26;81(3):654–66. doi: 10.1093/cid/ciae581 (PMC12497947; doi:10.1093/cid/ciae581)
Supplement: ciae581_Supplementary_Data [file ciae581_supplementary_data.docx]

**Supplementary Material**

**Noninferiority Study of Purified Vero Rabies Vaccine—Serum Free in 3-dose and 2-dose Preexposure Prophylaxis Regimens in Comparison With Licensed Rabies Vaccines**

Kulkanya Chokephaibulkit, Catherine Huoi, Terapong Tantawichien, Piroon Mootsikapun, Pope Kosalaraksa, Sasisopin Kiertiburanakul, Winai Ratanasuwan, Manuel Vangelisti, Thelma Laot, Youjun Huang, Celine Petit, Andrea-Clemencia Pineda-Peña, Carina Frago

**Supplementary Methods**

Ethical considerations

Inclusion and exclusion criteria

Randomization procedures

Vaccine administration

Sample size calculations

**Supplementary Tables**

Table S1. Sample size and estimated statistical power for the primary and key secondary immunogenicity objectives.

Table S2. Numbers of participants included in each analysis set by cohort and randomized group for primary vaccination series.

Table S3. RVNA titers with the three-dose PrEP primary series (Cohort 1, overall population, pooled children and adults) (PPAS for D42).

Table S4. RVNA titers with the two-dose PrEP primary series (Cohort 2, adults) (PPAS for D28).

Table S5. Solicited reactions within 7 days after any primary series vaccinations in Cohort 1 (three-dose, children and adults) and Cohort 2 (two-dose, adults) (SafAS).

**Supplementary Figures**

Figure S1. Participant flow through the study for the primary vaccination series.

Figure S2. Proportion of participants (95% CI) with RVNA titer ≥0.5 IU/mL with the two-dose PrEP primary series (Cohort 2, adults) (PPAS).

Figure S3. RVNA GMT (95% CI) with the two-dose PrEP primary series (Cohort 2, adults) (PPAS).

### Supplementary Methods

**Ethical considerations**

The study was conducted in accordance with the ethical principles of the Declaration of Helsinki and the International Conference on the Harmonisation - Good Clinical Practice. The study protocol and amendments were approved by the respective Institutional Review Boards (IRBs) or Independent Ethics Committees (IECs) at each study site. The study was later extended at one site with the approval of the IRB and IEC for this site. Written informed consent was obtained from all participants or their parents/legally acceptable representative before enrollment.

**Inclusion and exclusion criteria**

Participants were eligible for the study if they had not been bitten by or exposed to a potentially rabid animal in the previous 6 months and were not at high risk for rabies exposure during the study (e.g., animal handlers, laboratory workers).

Other exclusion criteria included: any vaccination in the 4 weeks preceding the first study vaccination or following any study vaccinations; receipt of immunoglobulins, blood, or blood-derived products in the past 3 months; known or suspected congenital or acquired immunodeficiency; immunosuppressive therapy in the past 6 months; known systemic hypersensitivity to any of the study or control vaccine components; bleeding disorder or anticoagulant therapy in the past 3 weeks, or self-reported thrombocytopenia; pregnancy or lactation; history of Guillain-Barré syndrome; acute illness/infection or febrile illness on the day of vaccination.

**Randomization procedures**

Separate randomization listings were created for each cohort. Participants were sequentially assigned an enrollment number, and randomly assigned to a study group by an interactive response technology system, using the permuted block method with a flexible block size of 10 and 20. The block size of 20 was used to accelerate the randomization for the booster phase adult subset at the beginning of enrollment. After the valid randomized number of adult participants receiving the booster reached the expected number, the block containing treatment groups without booster (block size of 10) was used to randomize the rest of the enrolled participants. Randomization was stratified by center for both cohorts; participants in Cohort 1 were also stratified by age group (children [1 year to <12 years and ≥12 to <18 years] and adult [≥18 years]).

**Vaccine administration**

Unblinded staff members at each site, who were independent of the safety evaluation and other study evaluations, prepared and administered the assigned vaccine. Blinded staff members and participants and their parents/legally acceptable representatives remained blinded for Cohort 1 and the primary vaccination series of Cohort 2 and did not know which vaccine was administered.

**Sample size calculations**

An alpha level of 2.5% (one-sided hypothesis), was chosen to calculate the sample size. Multiplicity adjustment for alpha was not applicable because the primary objective and five secondary immunogenicity objectives were tested sequentially.

Initial sample size calculations were based on an estimated attrition rate of 15% in the per-protocol analysis set (PPAS), assuming a proportion of participants with an RVNA titer ≥0.5 IU/mL of 99% for PVRV-NG2 and the control vaccines in each age group at D42. Thus, 303 participants in the PVRV-NG2 group and 101 participants in each of the control vaccine groups were required to be enrolled in each age group in Cohort 1. However, based on the results from another study (NCT03700242), the estimation of the proportion of adults with an RVNA titer ≥0.5 IU/mL at D28 was adjusted from 99% to 96.5% for PVRV-NG2 and the control vaccines, leading to the enrollment of 690 additional adults in Cohort 2. In addition, based on live blinded data from Cohort 1, the estimated attrition rates in Cohort 1 were adjusted to approximately 13.5% at both day (D) 28 and D42 for children and approximately 21.0% at D28 and 19.8% at D42 for adults, respectively. The attrition rate of adults in Cohort 2 was assumed to be similar to that of Cohort 1. The updated estimated number of evaluable participants and power calculations for each of the primary and key secondary objectives are shown in **Supplementary Table S1**.

Supplementary Table S1. Sample size and estimated statistical power for the primary and key secondary immunogenicity objectives.

| **Immunogenicity objective** | **Age group and cohort** | **Evaluable N (PPAS)** | **Estimation/margin** | **Power (%)** |
| --- | --- | --- | --- | --- |
| **Primary objective** | Children  Cohort 1 | PVRV-NG2: 261  PVRV: 88 | Participants with RVNA ≥0.5 IU/mL at D42: 99.0%  NI margin: -5% | 95.4^a^ |
|  |  | PVRV-NG2: 261  HDCV: 88 | Participants with RVNA ≥0.5 IU/mL at D42: 99.0%  NI margin: -5% | 95.4^a^ |
|  | Adults  Cohort 1 | PVRV-NG2: 243  PVRV: 81 | Participants with RVNA ≥0.5 IU/mL at D42: 99.0%  NI margin: -5% | 93.8^a^ |
|  |  | PVRV-NG2: 243  HDCV: 81 | Participants with RVNA ≥0.5 IU/mL at D42: 99.0%  NI margin: -5% | 93.8^a^ |
|  | **Overall: 80.0^a^** | | | |
| **Secondary objective #1** | Children and adults (pooled)  Cohort 1 | PVRV-NG2: 504 | Sufficiency threshold: Participants with RVNA ≥0.5 IU/mL at D42 ≥99.0%, LL of 95%CI ≥97.0% | 86.3^b^ |
| **Secondary objective #2** | Children  Cohort 1 | PVRV-NG2: 261  PVRV: 88 | Participants with RVNA ≥0.5 IU/mL at D28: 99.0%  NI margin: -5% | 95.4^a^ |
|  |  | PVRV-NG2: 261  HDCV: 88 | Participants with RVNA ≥0.5 IU/mL at D28: 99.0%  NI margin: -5% | 95.4^a^ |
|  | Adults  Pooled cohort 1 + 2 | PVRV-NG2: 570  PVRV: 190 | Participants with RVNA ≥0.5 IU/mL at D28: 96.5%  NI margin: -5% | 93.8^a^ |
|  |  | PVRV-NG2: 570  HDCV: 190 | Participants with RVNA ≥0.5 IU/mL at D28: 96.5%  NI margin: -5% | 93.8^a^ |
|  | **Overall: 80.1**^a^ | | | |
| **Secondary objective #3** | Children  Cohort 1 | PVRV-NG2 at D28: 261  HDCV at D42: 88 | Participants with RVNA ≥0.5 IU/mL at D28: 99.0%  Participants with RVNA ≥0.5 IU/mL at D42: 99.0%  NI margin: -10% | >99.9^a^ |
|  | Adults  D28: Pooled Cohort 1 + 2  D42: Cohort 1 | PVRV-NG2 at D28: 570  HDCV at D42: 81 | Participants with RVNA ≥0.5 IU/mL at D28: 96.5%  Participants with RVNA ≥0.5 IU/mL at D42: 99.0%  NI margin: -10% | >99.9^a^ |
|  | **Overall: >99.9**^a^ | | | |
| **Secondary objective #4** | Children and adults  Pooled Cohort 1 + 2 | PVRV-NG2: 831 | Sufficiency threshold: Participants with RVNA ≥0.5 IU/mL at D42 ≥99.0%, with LL of 95%CI ≥97.0% | **98.9^b^** |
| **Secondary objective #5** | Children and adults (pooled)  Cohort 1 | HDCV at D28: 168  HDCV at D42: 168 | Participants with RVNA ≥0.5 IU/mL at D28: 96.5%  Participants with RVNA ≥0.5 IU/mL at D42: 99.0%  NI margin: -10% | **90.0 ^c^** |

CI, confidence interval; D, day; HDCV, human diploid cell vaccine; LL, lower limit; N, number of participants; NI, non-inferiority; PVRV, purified Vero cell rabies vaccine; PVRV-NG2, next-generation purified Vero cell rabies vaccine; RVNA, rabies virus neutralizing antibody.

^a^Power calculated using Farrington and Manning method.

^b^Power calculated using binomial exact method.

^c^Power calculated using simulation (10,000 times, assuming correction coefficient =0.5), based on general linear model for repeated measured data with categorical response under binomial distribution.

Supplementary Table S2. Numbers of participants included in each analysis set by cohort and randomized group for primary vaccination series.

|  |  | **Cohort 1 (three-dose)** | | | **Cohort 2 (two-dose)** | | |
| --- | --- | --- | --- | --- | --- | --- | --- |
| **Primary series vaccine** |  | **Group 1 PVRV-NG2** | **Group 2 PVRV** | **Group 3 HDCV** | **Group 4 PVRV-NG2** | **Group 5 PVRV** | **Group 6 HDCV)** |
| **Primary series** | **N** | **607** | **203** | **200** | **420** | **139** | **139** |
| Randomized | Overall | 607 | 203 | 200 | 420 | 139 | 139 |
|  | Adults (≥18 years) | 302 | 103 | 100 | 420 | 139 | 139 |
|  | Children (<18 years) | 305 | 100 | 100 | 0 | 0 | 0 |
| FAS | Overall | 607 | 202 | 200 | 419 | 139 | 139 |
|  | Adults (≥18 years) | 302 | 103 | 100 | 419 | 139 | 139 |
|  | Children (<18 years) | 305 | 99 | 100 | 0 | 0 | 0 |
| FASI | Overall | 553 | 178 | 173 | 361 | 122 | 130 |
|  | Adults (≥18 years) | 267 | 88 | 82 | 361 | 122 | 130 |
|  | Children (<18 years) | 286 | 90 | 91 | 0 | 0 | 0 |
| PPAS D42 | Overall | 519 | 169 | 162 | 0 | 0 | 0 |
|  | Adults (≥18 years) | 254 | 84 | 79 | 0 | 0 | 0 |
|  | Children (<18 years) | 265 | 85 | 83 | 0 | 0 | 0 |
| PPAS D28 | Overall | 519 | 169 | 160 | 342 | 120 | 124 |
|  | Adults (≥18 years) | 253 | 83 | 79 | 342 | 120 | 124 |
|  | Children (<18 years) | 266 | 86 | 81 | 0 | 0 | 0 |
| SafAS | Overall | 607 | 202 | 200 | 419 | 139 | 139 |
|  | Adults (≥18 years) | 302 | 103 | 100 | 419 | 139 | 139 |
|  | Children (<18 years) | 305 | 99 | 100 | 0 | 0 | 0 |

FAS, full analysis set; FASI, FAS for immunogenicity; HDCV, human diploid cell vaccine; N, number of randomized participants; PPAS, per-protocol analysis set; PVRV, purified Vero cell rabies vaccine; PVRV-NG2, next-generation purified Vero cell rabies vaccine; SafAS, safety analysis set.

Supplementary Table S3. RVNA titers with the three-dose PrEP primary series (Cohort 1, overall population, pooled children and adults) (PPAS for D42).

|  | **Group 1**  **PVRV-NG2 (N=519)** | | **Group 2**  **PVRV (N=169)** | | **Group 3**  **HDCV (N=162)** | |
| --- | --- | --- | --- | --- | --- | --- |
| Participants with RVNA titer ≥0.5 IU/mL, | n/M | % (95% CI) | n/M | % (95% CI) | n/M | % (95% CI) |
| D0 | 0/519 | 0.0 (0.0; 0.7) | 0/169 | 0.0 (0.0; 2.2) | 0/162 | 0.0 (0.0; 2.3) |
| D28 | 512/512 | 100.0 (99.3; 100) | 167/168 | 99.4 (96.7; 100) | 158/160 | 98.8 (95.6; 99.8) |
| D42 | 519/519 | 100.0 (99.3; 100) | 168/169 | 99.4 (96.7; 100) | 162/162 | 100 (97.7; 100) |
| RVNA titer, IU/mL | GMT | (95% CI) | GMT | (95% CI) | GMT | (95% CI) |
| D0 | 0.100 | (0.100; 0.100) | 0.101 | (0.100; 0.102) | 0.101 | (0.100; 0.101) |
| D28 | 7.16 | (6.66; 7.69) | 4.90 | (4.31; 5.57) | 5.13 | (4.48; 5.87) |
| D42 | 24.0 | (22.4; 25.7) | 20.0 | (17.6; 22.8) | 16.4 | (14.7; 18.3) |
| RVNA titer, IU/mL | Median | IQR | Median | IQR | Median | IQR |
| D0 | 0.100 | 0.100; 0.100 | 0.100 | 0.100; 0.100 | 0.100 | 0.100; 0.100 |
| D28 | 7.97 | 4.47; 11.3 | 5.77 | 2.81; 8.30 | 6.11 | 2.86; 9.24 |
| D42 | 22.0 | 13.2; 42.2 | 18.9 | 11.4; 34.5 | 16.3 | 10.3; 26.7 |
| RVNA titer, IU/mL | Minimum | Maximum | Minimum | Maximum | Minimum | Maximum |
| D0 | 0.100 | 0.141 | 0.100 | 0.173 | 0.100 | 0.173 |
| D28 | 0.693 | 149.0 | 0.100 | 47.3 | 0.300 | 37.7 |
| D42 | 2.65 | 316.0 | 0.100 | 264.0 | 1.51 | 96.8 |

CI, confidence interval; D, day; GMT, geometric mean titer; HDCV, human diploid cell vaccine; IQR, interquartile range; M, number of participants with available data for the endpoint; N, number of participants in D42 PPAS; PPAS, per-protocol analysis set; PrEP, pre-exposure prophylaxis; PVRV, purified Vero cell rabies vaccine; PVRV-NG2, next-generation purified Vero cell rabies vaccine; RVNA, rabies virus neutralizing antibody.

Supplementary Table S4. RVNA titers with the two-dose PrEP primary series (Cohort 2, adults) (PPAS for D28).

|  | **Group 4**  **PVRV-NG2 (N=342)** | | **Group 5**  **PVRV (N=120)** | | **Group 6**  **HDCV (N=124)** | |
| --- | --- | --- | --- | --- | --- | --- |
| Participants with RVNA titer ≥0.5 IU/mL, | n/M | % (95% CI) | n/M | % (95% CI) | n/M | % (95% CI) |
| D0 | 0/342 | 0.0 (0.0; 1.1) | 0/120 | 0.0 (0.0; 3.0) | 0/124 | 0.0 (0.0; 2.9) |
| D28 | 332/342 | 97.1 (94.7; 98.6) | 118/120 | 98.3 (94.1; 99.8) | 119/124 | 96.0 (90.8; 98.7) |
| RVNA titer, IU/mL | GMT | (95% CI) | GMT | (95% CI) | GMT | (95% CI) |
| D0 | 0.101 | (0.100; 0.101) | 0.101 | (0.100; 0.101) | 0.100 | (0.100; 0.101) |
| D28 | 3.79 | (3.42; 4.20) | 2.92 | (2.45; 3.48) | 3.91 | (3.25; 4.70) |
| RVNA titer, IU/mL | Median | IQR | Median | IQR | Median | IQR |
| D0 | 0.100 | 0.100; 0.100 | 0.100 | 0.100; 0.100 | 0.100 | 0.100; 0.100 |
| D28 | 4.20 | 2.10; 6.45 | 2.84 | 1.53; 5.83 | 4.05 | 2.35; 7.48 |
| RVNA titer, IU/mL | Minimum | Maximum | Minimum | Maximum | Minimum | Maximum |
| D0 | 0.100 | 0.173 | 0.100 | 0.141 | 0.100 | 0.173 |
| D28 | 0.173 | 117.0 | 0.245 | 80.2 | 0.100 | 52.1 |

CI, confidence interval; D, day; GMT, geometric mean titer; HDCV, human diploid cell vaccine; IQR, interquartile range; M, number of participants with available data for the endpoint; N, number of participants in D28 PPAS; PPAS, per-protocol analysis set; PrEP, pre-exposure prophylaxis; PVRV, purified Vero cell rabies vaccine; PVRV-NG2, next-generation purified Vero cell rabies vaccine; RVNA, rabies virus neutralizing antibody.

Supplementary Table S5. Solicited reactions within 7 days after any primary series vaccinations in Cohort 1 (three-dose, children and adults) and Cohort 2 (two-dose, adults) (SafAS).

| **Participants in Cohort 1 experiencing  at least one event** | **Group 1**  **Three-dose** **PVRV-NG2** **(N=607)** | | | **Group 2**  **Three-dose** **PVRV**  **(N=202)** | | | **Group 3**  **Three-dose** **HDCV**  **(N=200)** | | |
| --- | --- | --- | --- | --- | --- | --- | --- | --- | --- |
|  | **n/M** | **%** | **(95%** **CI)** | **n/M** | **%** | **(95%** **CI)** | **n/M** | **%** | **(95%** **CI)** |
| Solicited reaction within 7 days after any vaccine injections in the primary series | 406/607 | 66.9 | (63.0; 70.6) | 118/201 | 58.7 | (51.6; 65.6) | 126/200 | 63.0 | (55.9; 69.7) |
| Solicited injection site reaction | 348/607 | 57.3 | (53.3; 61.3) | 98/201 | 48.8 | (41.7; 55.9) | 107/200 | 53.5 | (46.3; 60.6) |
| Tenderness/pain | 344/607 | 56.7 | (52.6; 60.7) | 95/201 | 47.3 | (40.2; 54.4) | 106/200 | 53.0 | (45.8; 60.1) |
| Erythema | 24/607 | 4.0 | (2.5; 5.8) | 6/201 | 3.0 | (1.1; 6.4) | 6/200 | 3.0 | (1.1; 6.4) |
| Swelling | 14/607 | 2.3 | (1.3; 3.8) | 4/201 | 2.0 | (0.5; 5.0) | 13/200 | 6.5 | (3.5; 10.9) |
| Solicited systemic reaction | 313/607 | 51.6 | (47.5; 55.6) | 88/201 | 43.8 | (36.8; 50.9) | 93/200 | 46.5 | (39.4; 53.7) |
| Fever | 50/606 | 8.3 | (6.2; 10.7) | 11/201 | 5.5 | (2.8; 9.6) | 4/200 | 2.0 | (0.5; 5.0) |
| Vomiting | 4/17 | 23.5 | (6.8; 49.9) | 1/2 | 50.0 | (1.3; 98.7) | 1/2 | 50.0 | (1.3; 98.7) |
| Crying abnormal | 8/17 | 47.1 | (23.0; 72.2) | 0/2 | 0 | (0; 84.2) | 1/2 | 50.0 | (1.3; 98.7) |
| Drowsiness | 4/17 | 23.5 | (6.8; 49.9) | 1/2 | 50.0 | (1.3; 98.7) | 0/2 | 0 | (0; 84.2) |
| Appetite lost | 3/17 | 17.6 | (3.8; 43.4) | 1/2 | 50.0 | (1.3; 98.7) | 0/2 | 0 | (0; 84.2) |
| Irritability | 8/17 | 47.1 | (23.0; 72.2) | 1/2 | 50.0 | (1.3; 98.7) | 0/2 | 0 | (0; 84.2) |
| Headache | 128/590 | 21.7 | (18.4; 25.2) | 49/199 | 24.6 | (18.8; 31.2) | 46/198 | 23.2 | (17.5; 29.7) |
| Malaise | 161/590 | 27.3 | (23.7; 31.1) | 45/199 | 22.6 | (17.0; 29.1) | 48/198 | 24.2 | (18.4; 30.8) |
| Myalgia | 249/590 | 42.2 | (38.2; 46.3) | 66/199 | 33.2 | (26.7; 40.2) | 78/198 | 39.4 | (32.5; 46.6) |
| **Participants in Cohort 2 experiencing  at least one event** | **Group 4**  **Two-dose PVRV-NG2  (N=419)** | | | **Group 5**  **Two-dose PVRV  (N=139)** | | | **Group 6**  **Two-dose HDCV  (N=139)** | | |
|  | **n/M** | **%** | **(95%** **CI)** | **n/M** | **%** | **(95%** **CI)** | **n/M** | **%** | **(95%** **CI)** |
| Solicited reaction within 7 days after any vaccine injections in the primary series | 128/419 | 30.5 | (26.2; 35.2) | 33/139 | 23.7 | (16.9; 31.7) | 47/139 | 33.8 | (26.0; 42.3) |
| Solicited injection site reaction | 111/419 | 26.5 | (22.3; 31.0) | 30/139 | 21.6 | (15.1; 29.4) | 43/139 | 30.9 | (23.4; 39.3) |
| Pain | 111/419 | 26.5 | (22.3; 31.0) | 30/139 | 21.6 | (15.1; 29.4) | 43/139 | 30.9 | (23.4; 39.3) |
| Erythema | 0/419 | 0 | (0; 0.9) | 0/139 | 0 | (0; 2.6) | 1/139 | 0.7 | (0; 3.9) |
| Swelling | 1/419 | 0.2 | (0; 1.3) | 0/139 | 0 | (0; 2.6) | 1/139 | 0.7 | (0; 3.9) |
| Solicited systemic reaction | 94/419 | 22.4 | (18.5; 26.7) | 16/139 | 11.5 | (6.7; 18.0) | 33/139 | 23.7 | (16.9; 31.7) |
| Fever | 2/419 | 0.5 | (0.1; 1.7) | 0/139 | 0 | (0; 2.6) | 0/139 | 0 | (0; 2.6) |
| Headache | 39/419 | 9.3 | (6.7; 12.5) | 9/139 | 6.5 | (3.0; 11.9) | 8/139 | 5.8 | (2.5; 11.0) |
| Malaise | 37/419 | 8.8 | (6.3; 12.0) | 10/139 | 7.2 | (3.5; 12.8) | 12/139 | 8.6 | (4.5; 14.6) |
| Myalgia | 78/419 | 18.6 | (15.0; 22.7) | 12/139 | 8.6 | (4.5; 14.6) | 29/139 | 20.9 | (14.4; 28.6) |

CI, confidence interval; HDCV, human diploid cell vaccine; M, number of participants with available data for the relevant endpoint; n, number of participants experiencing the endpoint listed; N, number of participants in SafAS; PVRV, purified Vero cell rabies vaccine; PVRV-NG2, next-generation purified Vero cell rabies vaccine; SafAS, safety analysis set.

Percentages are based on M of each age group.

Supplementary Figure S1. Participant flow through the study for the primary vaccination series.


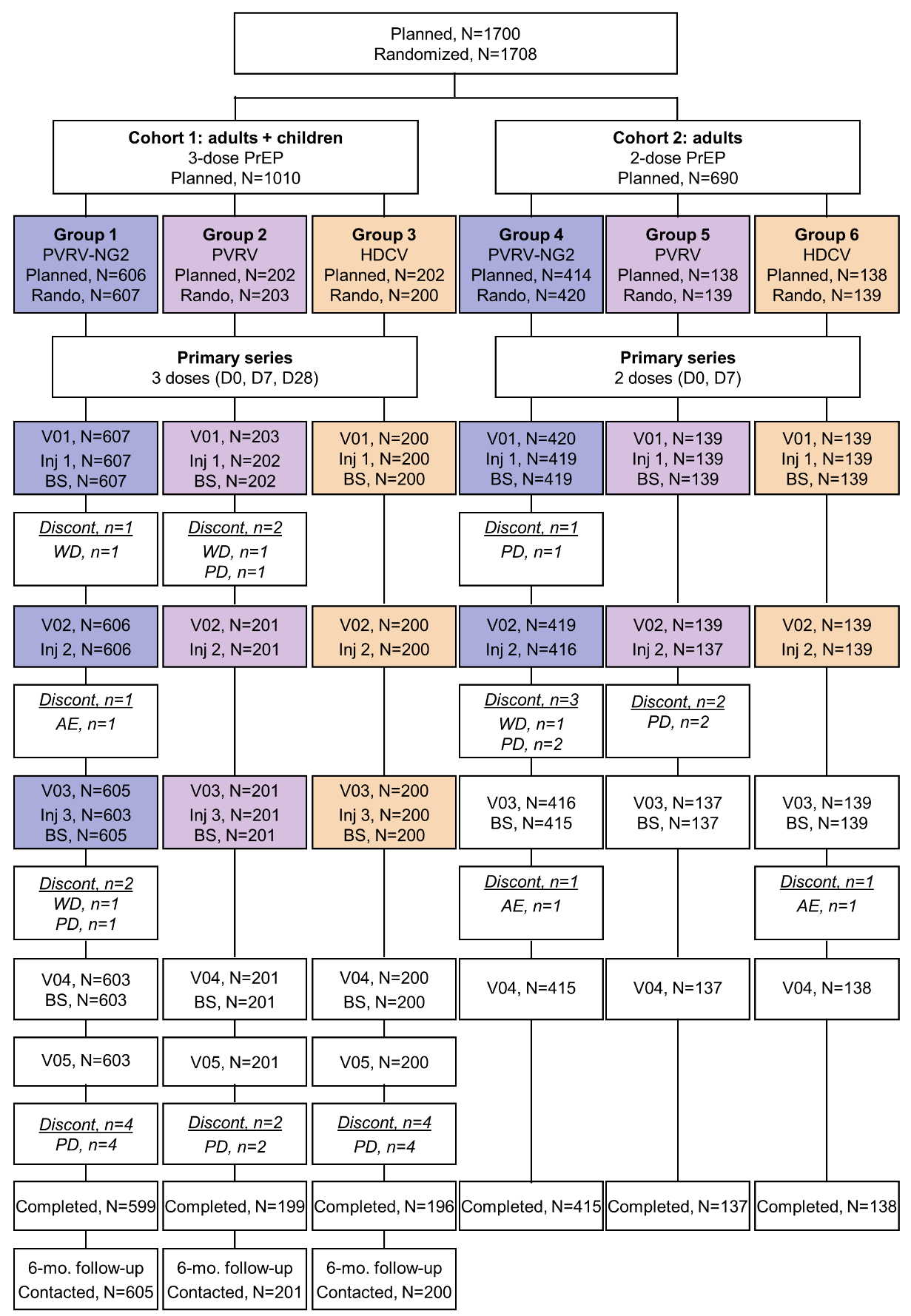


AE, adverse event; BS, blood sample; D, Day; Discont, discontinued; HDCV, human diploid cell vaccine; Inj, injection; Mo, month; PD, protocol deviation; PrEP, pre-exposure prophylaxis; PVRV, purified Vero cell rabies vaccine; PVRV-NG2, next-generation purified Vero cell rabies vaccine; rando, randomized; V, Visit; WD, withdrawal.

A follow-up phone call was scheduled 6 months after the primary series for all participants in Cohort 1 and for participants not included in the immune persistence and booster phase subset of Cohort 2.

Supplementary Figure S2. Proportion of participants (95% CI) with RVNA titer ≥0.5 IU/mL with the two-dose PrEP primary series (Cohort 2, adults) (PPAS).


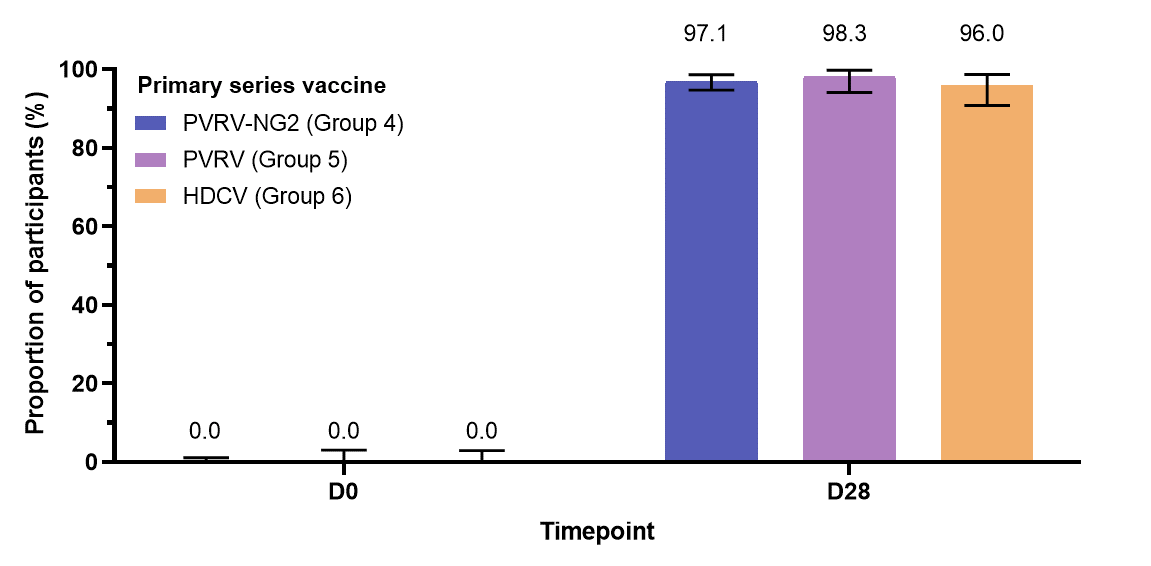


CI, confidence interval; D, day; HDCV, human diploid cell vaccine; PPAS, per-protocol analysis set; PrEP, pre-exposure prophylaxis; PVRV, purified Vero cell rabies vaccine; PVRV-NG2, next-generation purified Vero cell rabies vaccine; RVNA, rabies virus neutralizing antibody.

Supplementary Figure S3. RVNA GMT (95% CI) with the two-dose PrEP primary series (Cohort 2, adults) (PPAS).


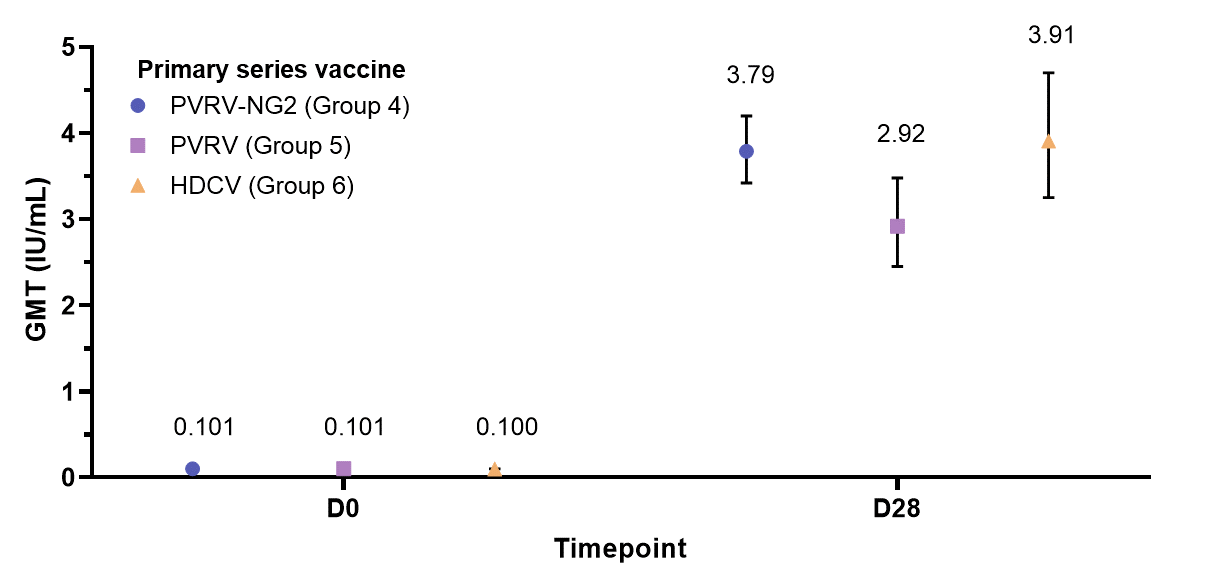


CI, confidence interval; D, day; GMT, geometric mean titer; HDCV, human diploid cell vaccine; PPAS, per-protocol analysis set; PrEP, pre-exposure prophylaxis; PVRV, purified Vero cell rabies vaccine; PVRV-NG2, next-generation purified Vero cell rabies vaccine; RVNA, rabies virus neutralizing antibody.
